# High-Impact Performance and Thermal Properties of Polyimine Nanocomposites Reinforced by Silicon Carbide Nano-Whiskers

**DOI:** 10.3390/ma16134587

**Published:** 2023-06-25

**Authors:** Shiyu Ji, Si Zhang, Zifan Wang, Chaoyue Li, Wenjing Cao, Yongmei Zhu, Chaoshuai He, Yun Chen

**Affiliations:** 1 School of Mechanical Engineering, Jiangsu University of Science and Technology, Zhenjiang 212100, China; jsy932664879@163.com (S.J.); wang.zifan@foxmail.com (Z.W.); njzybest707@163.com (W.C.); zymtt@163.com (Y.Z.); chaoshuai98@163.com (C.H.); 2 China Tianchen Engineering Corporation, Tianjin 300400, China; leechaoyue@gmail.com

**Keywords:** mechanical properties, polyimine, silicon carbide nano-whiskers, nanocomposites, thermal stability

## Abstract

Polymer nanocomposites, which combine the advantages of polymers and fillers, are widely used in the field of automobile and aviation. Polyimine (PI) is an emerging thermoset material with remarkable properties, such as malleability, recyclability, and self-healing. Silicon carbide nano-whiskers (SiCw), as a cheap and high-hardness filler material, are chosen to enhance the properties of polyimine matrix. Silicon carbide nano-whisker-reinforced polyimine (PI-SiCw) nanocomposites were successfully fabricated by heat pressing, which was confirmed by FTIR and XPS tests. According to the results of mechanical tests, the mechanical properties of PI-SiCw nanocomposites were obviously improved. For example, with the addition of 0.5% SiCw, bending strength and bending elongation at break can be simultaneously increased by 33% and 148%, respectively. Surprisingly, the impact strength of PI-SiCw nanocomposites with 2% SiCw was increased by 154% compared to the matrix. SEM and EDS tests showed that the evenly distributed SiCw in the polyimine matrix enhanced the mechanical properties of PI-SiCw nanocomposites according to the mechanism of whiskers pulling out and the bridging principle. According to the TGA test results, the PI composites with SiCw retain a higher weight percentage at 800 °C. The reason was the combined effect of the good thermal stability of SiCw and their strong interactions with the PI matrix. As a result, introducing SiCw into the PI matrix imparts a slight improvement in thermal stability. This article presents an avenue of cost-effective research to enhance the mechanical properties of polyimine composites.

## 1. Introduction

Polymer nanocomposites exhibit excellent mechanical and thermal properties, combining the advantages of polymers and nano-fillers. In recent years, embedding inorganic fillers as reinforcing phases into a polymer matrix has become the focus of research [1,2,3,4,5]. A variety of inorganic nano-fillers such as silicon carbide, graphene, and zirconia were used to fabricate polymer composites. Zhang et al. found that the toughness of polyimine enhanced by zirconia nanoparticles can be increased by 85% [6]. Du et al. discovered that the overall performance of silicone rubber was improved by adding nanosilica [7]. Li et al. found that the synergy between BN and CNT contributes to the formation of a three-dimensional filler network, which improves the mechanical properties of fluororubber [8]. Mekonen et al. proved that the maximum tensile strength and compressive strength of epoxy resin composites can be greatly enhanced by mixing 40% e-glass fiber into a 60% epoxy resin matrix [9]. Mingchao et al. discovered SiCw and CNTs showed optimum mechanical behavior when their content in the resin was 0.75 wt% and 0.3 wt%, respectively [10]. From these examples, it is obvious that low concentrations of inorganic nano-fillers have a good effect on improving the mechanical properties of polymer composites, which can meet a variety of industrial requirements.

Polyimine (PI), is an emerging thermoset material, prepared by condensation of aldehyde and amine [11,12,13,14,15] (C=N, dynamic–covalent interaction of imine bonds) under mild reaction conditions. Unlike typical thermoset materials, PI has many remarkable properties, such as malleability, recyclability, and self-healing [16,17,18,19]. However, the mechanical strength of PI cannot meet industrial needs. The mechanical and thermal properties of PI can be enhanced significantly without changing other original properties by adding nano-fillers, so the addition of nano-fillers to the matrix is an essential means of boosting the properties of composites.

Among the numerous reinforced fillers, silicon carbide (SiC) [20] is a high-hardness material. In order to obtain a better bonding effect with the reinforcing matrix, the surface size of the SiC particles is reduced to the nanometer scale. Nano-scale fibrous whiskers are able to grow along the crystal plane among the silicon carbide under the action of the catalyst [21]. Compared to silicon carbide, the strength of silicon carbide nano-whiskers (SiCw) was improved greatly [22]. Above all, the addition of SiCw is a better choice for enhancing the mechanical and thermal properties of PI composites [23,24].

In this work, PI was produced by mixing terephthalaldehyde (TA), diethylenetriamine (DETA), and triethylenetetramine (TETA). Polyimine with silicon carbide nano-whisker (PI-SiCw) nanocomposites was successfully prepared by heat pressing mixed PI powder and silicon carbide nano-whiskers. Mechanical tests of hardness, tensile strength, bending strength, and impact strength were conducted to analyze the mechanical properties enhancement of PI-SiCw nanocomposites. In addition, scanning electron microscopy (SEM) and energy dispersive spectroscopy (EDS) tests were employed to observe the fracture surfaces from distinct mechanical tests. The thermal stabilities and heat resistance of PI-SiCw nanocomposites were investigated by thermogravimetric analysis (TGA), derivative thermogravimetry (DTG), and differential scanning calorimetry (DSC). 

## 2. Materials and Methods

### 2.1. Experimental Material

Terephthalaldehyde, diethylenetriamine, and triethylenetetramine were purchased from Aladdin Industries (Shanghai, China), with a purity of 98%, 99%, and 99%, respectively; silicon carbide nano-whiskers were purchased from Shanghai Coconut Environmental Protection Technology Co., Ltd. (Shanghai, China), with a purity of >99.99%. All chemicals have not been further purified.

### 2.2. Experimental Process

PI was prepared by TA, DETA, and TAEA at a ratio of 3:0.9:1.4. The above chemicals were stirred in 60 °C ethyl acetate with no catalyst added in the process [25], then the precipitate gradually separated out in the process of mixing. The solid PI powder can be obtained after putting the precipitate in a drying box for drying treatment at 60 °C. The PI powder obtained was ground in a grinding machine and then sieved with an 80-mesh screen. Lastly, the PI powder required for this experiment could be prepared. The preparation method of PI-SiCw nanocomposites was as follows: the finished PI powder was placed in the drying box to dry at 60 °C for 1 h. Silicon carbide nano-whiskers with different content were then mixed with the PI powder by a YXQM planetary grinding machine at a speed of 100 r/min for 1 h. The PI-SiCw nanocomposites were prepared by heat pressing at 80 °C under 9 MPa for 1 h.

### 2.3. Characterization

The hardness test was performed on an HBRVS-18T7.5 Digital Display Brinell hardness tester (Laizhou, China). The HRR model was used for the test, together with a ball indenter with a diameter of 12.70 mm. The test samples were rectangular samples (35mm × 5mm × 4mm). 

The tensile strength and bending strength tests were performed on a WDW-005 type microcomputer-controlled electronic universal testing machine (Jinan Hengsi Shanda Instruments Co., Ltd.) (Jinan, China). The test speed for both tensile and bending tests was 0.1 mm/min. The effective size for the tensile strength test was 5mm × 2mm × 2mm. The effective for the bending strength test was 35mm × 5mm × 4mm.

The XJJ-50S-type liquid crystal display simple beam impact tester (Jinan Hengsi Shanda Instruments Co., Ltd.) (Jinan, China) was adopted to test impact strength in this experiment. The pendulum energy was 7.5 J. The effective size for the impact strength test was 35mm × 5mm × 4mm.

A Fourier transform infrared (FTIR) spectroscopy test was performed on a Fourier infrared spectrometer produced by Thermo Fisher in the Waltham, MA, USA. Samples of PI and PI-SiCw nanocomposites were prepared by KBr compression. 

An X-ray photoelectron spectroscopy (XPS) test was performed on an X-ray photoelectron spectrometer produced by Thermo Fisher in the United States. Sample preparation of the PI and PI-SiCw nanocomposite powder was carried out by pressing (pressure: 5 MPa, time: 10 s).

Thermogravimetric analysis (TGA) and derivative thermogravimetry (DTG) were performed on a Q600 thermogravimetric analyzer produced by the TA Corporation in the United States. In the test, 3 mg samples were weighed. The temperature range was 30–800 °C and the heating rate was 10 °C/min. During the test, nitrogen was used for protection. 

Differential scanning calorimetry (DSC) analysis was performed on a differential scanning calorimeter produced by Mettler, Switzerland. In the test, 5 mg samples were weighed. The temperature range was controlled at 30–800 °C, the heating rate was 10 °C/min, and nitrogen was used for protection during the test.

The fracture of the PI and PI-SiCw nanocomposites was observed by scanning electron microscopy (SEM) (Hitachi S4800) (Tokyo, Japan). All samples were coated with gold before scanning electron microscopy. 

## 3. Results and Discussion

### 3.1. Preparation of PI-SiCw

**Figure 1:**
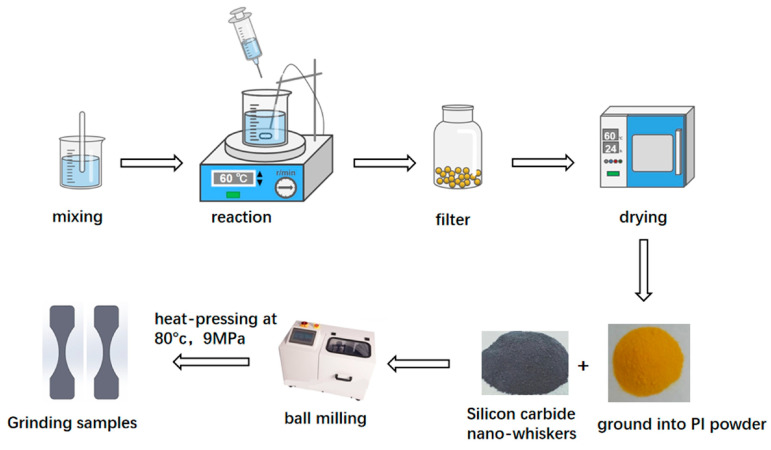
Manufacturing process of SiCw-reinforced PI composites.

**Figure 2:**
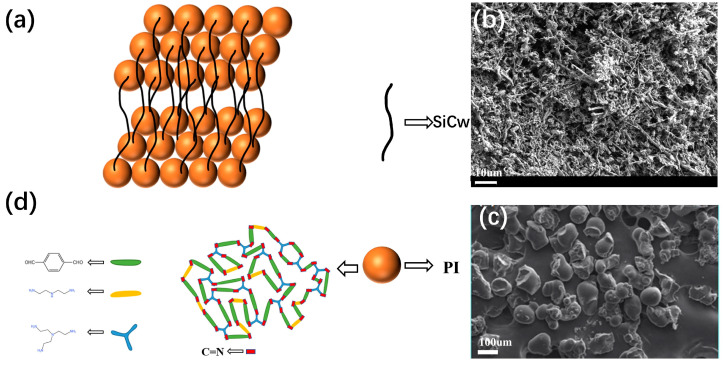
(**a**) Schematic illustration of the fabrication of PI-SiCw nanocomposites. SEM images of (**b**) silicon carbide nano-whiskers and (**c**) PI powder reproduced from ref. [6] with permission from John Wiley and Sons, copyright 2017. (**d**) Schematic illustration of the fabrication of PI.

The process of PI-SiCw produced by heat pressing is shown in Figure 1. Reasons for the successful preparation of PI-SiCw are presented in Figure 2. The aldehyde group on TA reacted with the primary amine group on DETA and TETA under the action of ethyl acetate according to the condensation of aldehyde and amine (Figure 2d). The covalent dynamics of the C=N double bond endowed the PI powder with reproducibility. Therefore, PI powder mixed with SiCw can be formed under the condition of heat pressing (Figure 2a). Samples of PI-SiCw nanocomposites prepared by heat pressing were named PI, PI-SiCw-0.5, PI-SiCw-1, PI-SiCw-2, and PI-SiCw-5, respectively, corresponding to the matrix with silicon carbide nano-whiskers content of 0%, 0.5%, 1%, 2%, and 5%, respectively.

**Figure 3:**
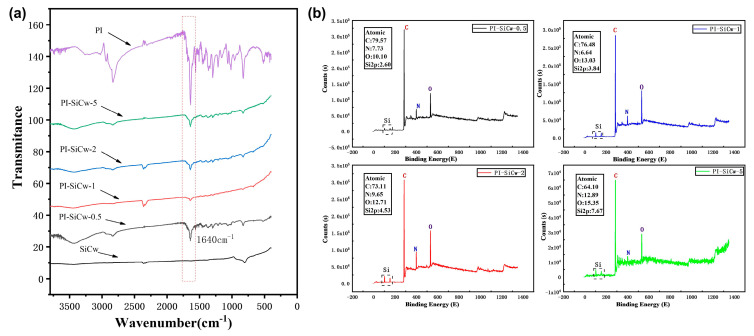
(**a**) FTIR spectra of PI and PI-SiCw nanocomposites, (**b**) Wide-scan survey XPS spectrum of PI-SiCw nanocomposites.

Figure 3a shows the FTIR spectra of SiCw, PI, and PI-SiCw with different content. TA aldehyde-based distinctive peaks at 1693 cm^−1^ in the polyimine-based infrared atlas disappeared, and the distinctive peak at around 1640 cm^−1^ triggered by C=N double bond (C=N stretching vibration) was consistent with the imine peak. Each curve of PI-SiCw-0.5, PI-SiCw-1, PI-SiCw-2, and PI-SiCw-5 had two distinctive peaks; one was near 1640 cm^−1^ caused by the C=N double bond stretching vibration, and the other was at around 800 cm^−1^ corresponding to the Si-C bond. In the curve of SiCw, the S-C bond was also observed at the position around 800 cm^−1^. Therefore, SiCw were successfully embedded into the matrix. 

XPS curves of PI-SiCw samples are shown in Figure 3b. The distinctive peaks at around 285.1 eV, 399.1 eV, and 532.1 eV confirmed the existence of carbon(C), nitrogen(N), and oxygen (O) elements, respectively. Furthermore, the distinctive peak of Si appeared at around 102.1 eV [26] after the addition of SiCw. From the distinctive peaks of C, N, O, and Si, it can be concluded that SiCw were incorporated into the matrix. In addition, from the curves of the XPS test, it can be seen that as the SiCw content increased, the proportion of the Si element in PI-SiCw nanocomposites continued to increase, indicating that PI-SiCw-0.5, PI-SiCw-1, PI-SiCw-2, and PI-SiCw-5 nanocomposites have been successfully prepared.

**Figure 4:**
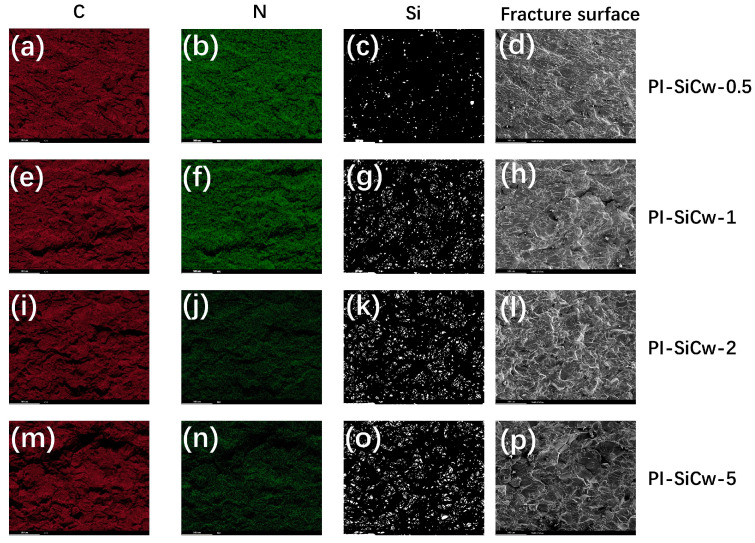
Elemental mappings of C (**a**,**e**,**i**,**m**), N (**b**,**f**,**j**,**n**) and Si (**c**,**g**,**k**,**o**) of PI-SiCw according to the fracture surface of PI-SiCw nanocomposites (**d**,**h**,**I**,**p**).

To observe the distribution of each element inside the samples, element mapping analysis was employed (Figure 4). C, N, and Si elements were all present in the PI-SiCw nanocomposites with different contents. Elements of C and N were relatively uniformly distributed, but the distribution of Si elements changed with the addition of various amounts of SiCw. From Figure 4o, it is obvious that irregular aggregation of SiCw occurred. The agglomeration of the whiskers reduces the bonding force, thus decreasing the mechanical properties.

### 3.2. Mechanical Properties

#### 3.2.1. Hardness and Tensile Strength Analysis

**Figure 5:**
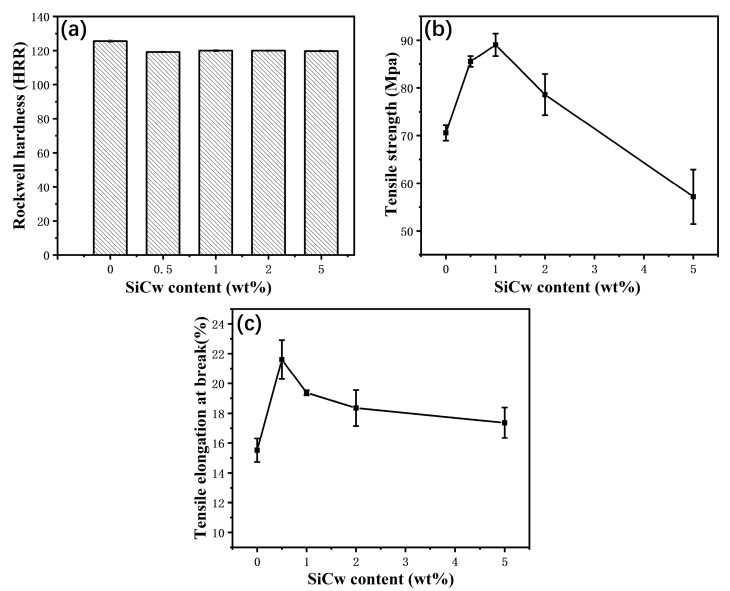
(**a**) Hardness and tensile properties of PI-SiCw nanocomposites: (**b**) tensile strength, (**c**) tensile elongation at the break.

Figure 5a presents the hardness of PI-SiCw nanocomposites with various amounts of added SiCw. The results suggest that both PI-SiCw nanocomposites and PI had high hardness around 120 HRR. This implies that PI- SiCw nanocomposites can still maintain high hardness in the range of a small amount of added SiCw. Figure 5b,c shows the curves of tensile strength and tensile elongation at break PI-SiCw nanocomposites with different amounts of added SiCw. The tensile strength curve of PI-SiCw shows a trend of increasing first and then decreasing. When the addition of SiCw was 1%, the tensile strength of PI-SiCw reached the maximum value (89.05 MPa), which was 26% higher than that of the matrix. Tensile elongation at the break of PI-SiCw rose by 39% from 15.52% for the matrix to 21.61% for PI-SiCw-0.5. However, when the SiCw content exceeded 1%, the tensile strength and tensile elongation at the break of PI-SiCw showed an obvious decline. Due to the extremely high plasticity and mechanical strength of SiCw, the tensile strength and tensile elongation at break were enhanced as a whole [27], which can withstand greater external tensile force. 

**Figure 6:**
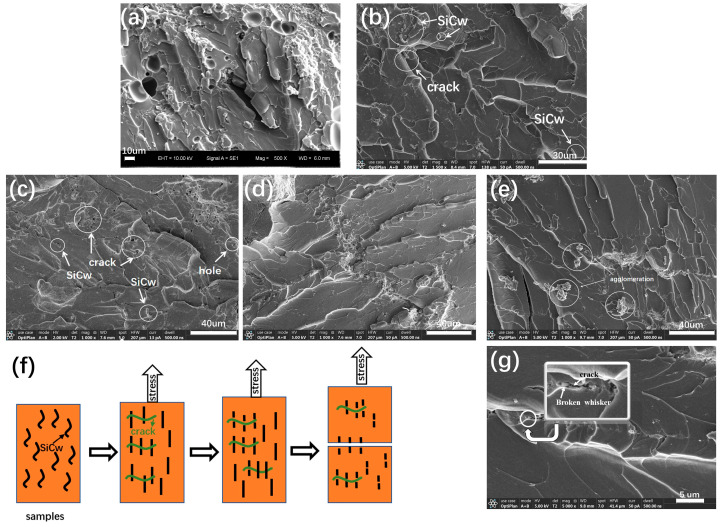
SEM images of tensile fracture surfaces of (**a**) PI and PI-SiCw nanocomposites: (**b**) PI-SiCw-0.5, (**c**) PI-SiCw-1, (**d**) PI-SiCw-2, (**e**) PI-SiCw-5. (**f**) Tensile resistance mechanism. (**g**) SEM images of broken whisker.

Figure 6a–e show SEM pictures of tensile fractures. Plenty of patterns were observed in SEM images, so it can be clearly seen that the fracture type of PI- SiCw nanocomposites belonged to brittle fractures. There are a few cracks in Figure 6b,c. The crack bent and turned along the interface of the matrix when meeting high-strength SiCw during the expansion process, thus prolonging the crack propagation path in the material and consuming more energy. Not only that, uniformly distributed SiCw (Figure 6b,c) expanded the filling area between the matrix and reinforcement phases, giving PI and SiCw better bonding force. Thus, the tensile strength of PI-SiCw-1 nanocomposites also reached the maximum value. The schematic diagram of the microscopic morphology of SiCw-enhanced PI during the fracture process of the tensile specimen is shown in Figure 6f. In the initial stage of stretching, the embedded SiCw was straightened, caused by a deformed sample, and the friction between SiCw and the matrix consumed energy. As the load increased, cracks occurred, and the internal SiCw acted as a bridge, generating force to prevent cracks from expanding. After the stress reached the limit value, the sample was pulled apart. It can be observed from the surface of the cross-section (Figure 6g) that the broken SiCw appeared near the crack. The energy required for crack propagation was consumed by the fracture of SiCw. Therefore, SiCw played a role in preventing crack propagation. However, When SiCw content was beyond 1%, a large number of whiskers agglomerated, resulting from uneven dispersion (Figure 6e) and causing stress concentration and reducing mechanical behaviors. This also explained the reason for both decreases in the tensile strength and tensile elongation at break of PI when the addition of SiCw was 5%.

#### 3.2.2. Bending Strength Analysis

**Figure 7:**
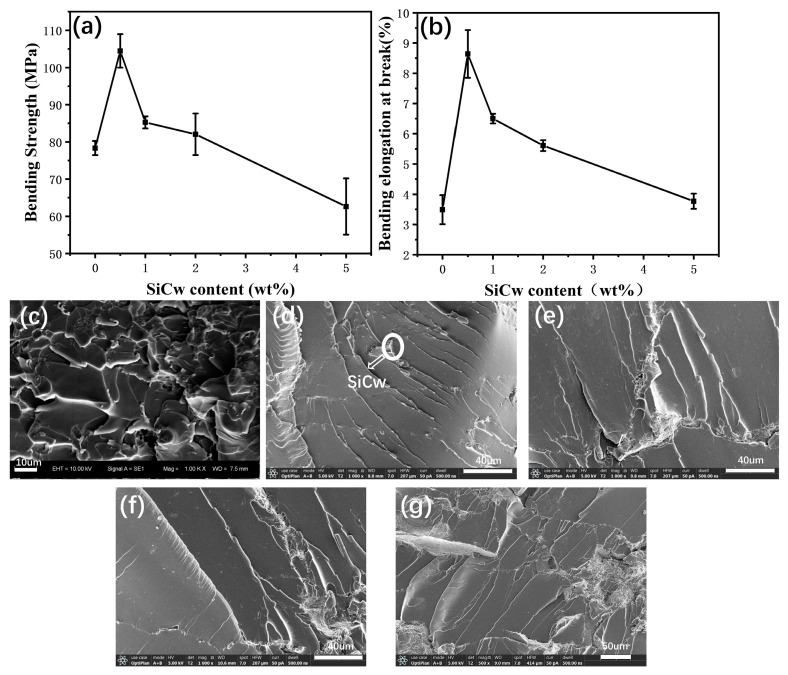
(**a**) Bending strength of PI and PI-SiCw nanocomposites (**b**) Bending elongation at break figures of PI and PI-SiCw nanocomposites. SEM images of bending fracture surfaces of (**c**) PI and PI-SiCw nanocomposites: (**d**) PI-SiCw-0.5, (**e**) PI-SiCw-1, (**f**) PI-SiCw-2, (**g**) PI-SiCw-5.

Figure 7 shows the bending strength curve of PI-SiCw nanocomposites. The bending strength of PI, PI-SiCw-0.5, PI-SiCw-1, PI-SiCw-2, and PI-SiCw-5 were 78.35 MPa, 104.45 MPa, 85.25 MPa, 82.05 MPa, and 62.65 MPa, respectively. With the increase in the content of SiCw, bending strength increased first and then decreased (Figure 7a). PI -SiCw-0.5 had the highest bending strength (104.45 Mpa), which was 33% higher than that of the matrix. Curves of bending elongation at break and bending strength had the same trend of change. The elongation at break of the PI-SiCw nanocomposite reached the extreme value with the addition of 0.5% SiCw (8.64%) (Figure 7b), which was increased by 148% compared to the matrix. Similar to the enhancement mechanism of mechanical stretching, evenly dispersed SiCw effectively interacted with the matrix [28,29], thereby enhancing the bending performance of PI- SiCw nanocomposites. Compared with the matrix (Figure 7c), the microstructure fracture of PI-SiCw-0.5 (Figure 6d) showed lots of river patterns, which were embedded by ridge lines produced by SiCw. Uniformly arranged nano-whiskers in ridge lines can absorb the energy of crack propagation via several roles [30], which can deflect stress and prevent the propagation of fracture cracks, making it suitable for improving bending strength.

#### 3.2.3. Impact Strength Analysis

**Figure 8:**
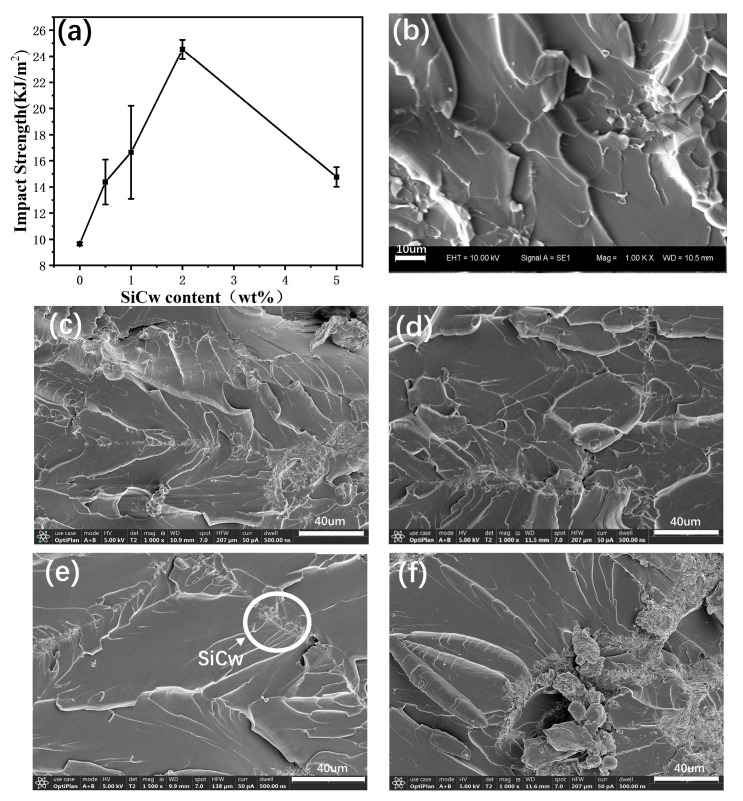
(**a**): Impact strength of PI and PI-SiCw nanocomposites. SEM images of bending fracture surfaces of (**b**) PI and PI-SiCw nanocomposites: (**c**) PI-SiCw-0.5, (**d**) PI-SiCw-1, (**e**) PI-SiCw-2, (**f**) PI-SiCw-5.

Figure 8 presents the impact strength of PI and PI-SiCw nanocomposites. The impact performance of PI was excellent overall after adding SiCw. The impact strength of PI, PI-SiCw-0.5, PI-SiCw-1, PI-SiCw-2, and PI-SiCw-5 was 9.66 KJ/m^2^, 14.37 KJ/m^2^, 16.65 KJ/m^2^, 24.52 KJ/m^2^ and 14.76 KJ/m^2^, respectively. Surprisingly, when the addition of SiCw was 2%, the impact properties of the matrix were significantly enhanced. Compared to the matrix, the impact strength of PI-SiCw-2 was increased by 154%. As shown in Figure 8e, some SiCw appeared around the cracks, bridging between cracks in the matrix and thereby creating the force to close the cracks [31,32]. Therefore, the nanocomposites can withstand greater external force impact, which was a core reason for the impact strength of the SiCw-reinforced matrix. Consistent with the results of tensile and bending testing, when the addition of SiCw was over 5%, the impact strength was greatly reduced because stress concentration points were formed in the case of the aggregation of large amounts of SiCw (Figure 8f), affecting the effective transfer of stress.

The mapping diagrams of the impact test section shown in Figure 4 were used to further analyze the mechanism of the enhancement of mechanical properties. The distribution of carbon (C) and nitrogen (N) elements in the matrix was relatively uniform. Similar to the fracture ridges observed in SEM images, with the gradual increase in SiCw, the irregular distribution of the Si element became more obvious (Figure 4o). The morphology formed by the irregular aggregation of SiCw was similar to the ridge morphology of the fracture. Especially for PI-SiCw-5, the elemental mapping of Si (Figure 4o) revealed an agglomerated pattern that resembled the surface cracks of the fracture observed in Figure 8f, which indicated that these ridges contained a large amount of Si element.

The mechanical properties of the PI can be enhanced with the introduction of SiCw according to the test results for tensile, bending, and impact strength (Table 1). The main reasons for the enhancement were as follows: The expansion of cracks can be prevented due to the interaction between SiCw and PI. The friction generated by SiCw under the action of the applied load consumed plenty of energy. As the load increased, SiCw acted as a bridge between cracks. However, for a specific mechanical property requirement, the amount of SiCw used to achieve the optimum may vary, resulting in various effects of property enhancement, which were determined by the test conditions. For example, SiCw were pulled out when they were subjected to tension during the tensile test, and holes were left in the fracture surface (Figure 6c), but the bending test and impact test did not have such results (Figure 7c–g and Figure 8b–f). In addition, the impact test samples had rougher fracture surfaces owing to higher impact loads compared to the bending test, so it was not surprising that the optimal performance exhibited with various amounts of added SiCw was different. 

From the above results, it can be seen that the addition of SiCw had a good effect on the mechanical strength of PI composites. These high-strength imine materials can be well adapted to industrial applications. Moreover, PI materials can be recycled due to the easy decomposition of imine bonds under weak acid conditions, indicating that PI-SiCw can have broad application prospects in the field of environmentally friendly materials [33,34].

In order to explore the advantages of PI-SiCw composites, additional work was undertaken regarding the comparative analysis. Compared to adding graphene sheets into the PI matrix, we found that adding SiCw can better improve the overall performance of PI [35]. In addition, compared to adding graphene, using SiCw as the reinforcing phase can save costs. Moreover, compared to traditional PVA materials [36], the PI-SiCw nanocomposites prepared in this work not only had better mechanical properties but can also be recycled and reprocessed. The high performance and good recyclability of PI-SiCw composites make them a promising environmentally friendly alternative to many conventional plastic products.

### 3.3. Thermal Properties

#### 3.3.1. TGA and DTG Analysis

**Figure 9:**
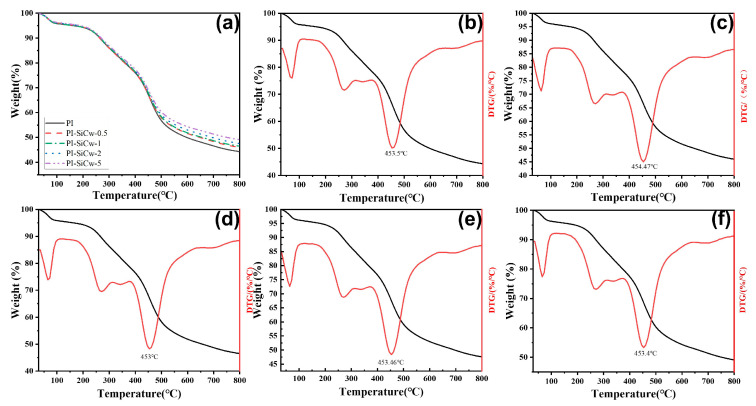
TGA and DTG curves of PI and PI-SiCw. (**a**) TGA curves of PI and PI-SiCw nanocomposites. (**b**)TG-DTG curve of PI, (**c**) TG-DTG curve of PI-SiCw-0.5, (**d**) TG-DTG curve of PI-SiCw-1, (**e**) TG-DTG curve of PI-SiCw-2, (**f**) TG-DTG curve of PI-SiCw-5.

The thermal stabilities of PI-SiCw nanocomposites were observed by TGA and DTG (Figure 9a–f). The results of loss rate measurements of PI-SiCw nanocomposites from 30 °C to 800 °C are shown in Figure 9a. DTG curves of PI-SiCw nanocomposites (Figure 9b–f) were derivatives of the thermogravimetric curves. Overall, the residual weight of PI-SiCw nanocomposites was larger than that of the matrix, which increased from 44% to 49%. Moreover, the mass loss rate of PI-SiCw nanocomposites was fast around 65 °C and 450 °C. In the process of heating, the mass loss detected by TGA ranging from 50 °C to 100 °C may be due to the evaporation of water and some latent decomposition of polymer composites by heating [37]. The reaction process was divided into two stages. Due to the opening of the internal functional groups of the material molecules under a temperature higher than 400 °C, the hydroxyl content was drastically reduced, thereby decreasing the weight of the material at a rapid rate. The weight loss trend continued to be gentle till 500 °C on account of the carbonization of the samples. In comparison to the matrix, PI-SiCw nanocomposites possessed a high residual weight percentage. For example, when the temperature was 500 °C, the residual mass fraction of the matrix was only 56.7%, while the residual mass fractions of PI-SiCw-0.5, PI-SiCw-1, PI-SiCw-2, and PI-SiCw-5 were 57.9%, 58.2%, 59.1%, and 60.3%, respectively. With the increase in SiCw, the residual mass of SiCw nanocomposites was higher than that of the matrix till 800 °C. It can be seen from the changes in the heat loss of PI-SiCw nanocomposites that the addition of SiCw can improve the thermal stability of nanocomposites. As shown in Figure 9b–f, the maximum decomposition rate temperature (T_max_) remained high as a whole. Interestingly, with the increase in SiCw content, the decomposition temperature at 50% weight loss (T_50%_) of PI-SiCw showed a more obvious upward trend. This also showed that the thermal stabilities of PI-SiCw were better than that of the matrix. The results implied that SiCw can increase the volume owing to high thermal conductivities, so the interfacial thermal resistance between the matrix and SiCw decreased, thereby improving the thermal stabilities of PI-SiCw nanocomposites [38,39] (Table 2).

#### 3.3.2. DSC Analysis

**Figure 10:**
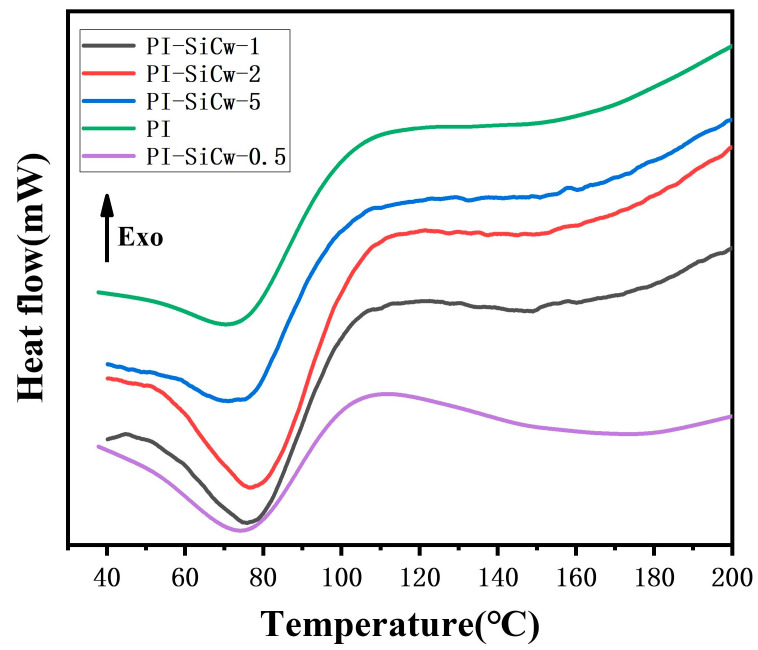
DSC curves of PI-SiCw nanocomposites of different SiCw content.

A differential scanning calorimetry (DSC) test was used to study the thermal performance of PI and PI-SiCw nanocomposites. With the increase in SiCw content, the temperature corresponding to the inflection point of the DSC curve will also increase. The accurate determination of Tg within the endothermic peak is difficult, and it seems that a minor increase in Tg may occur by increasing the SiCw content. The increase in Tg will protect PI-SiCw nanocomposites against melting under high temperatures (Figure 10).

Moreover, combined with TGA and DSC curves, it was found that when the temperature reached the softening temperature of PI and PI-SiCw, the nanocomposites had a certain amount of mass loss, which may be due to the fact that latent decomposition reached a maximum at the softening point [37]. This also explained the phenomenon of mass loss in PI-SiCw in the TGA curve before reaching a temperature of 100 °C.

Overall, introducing SiCw into the PI matrix can reduce the weight loss of PI-SiCw materials at high temperatures while also increasing the glass transition temperature of PI composites, favoring the heat resistance improvement of PI-SiCw nanocomposites. 

## 4. Conclusions

PI-SiCw nanocomposites can be successfully fabricated by heat pressing under mild conditions. Compared with the matrix, the prepared nanocomposites have significantly improved mechanical properties such as impact strength, toughness, tensile strength, and bending strength. With the addition of 0.5% SiCw, bending strength, tensile elongation at break, and bending elongation at break of PI-SiCw nanocomposites were greatly increased by 33%, 39%, and 148%, respectively. When the addition of SiCw was 1%, the tensile strength of PI-SiCw nanocomposites was enhanced by 26%. Interestingly, after adding 2% SiCw, the impact strength of PI-SiCw nanocomposites was increased by 154%. The main reasons for the enhanced mechanical properties of PI-SiCw nanocomposites are crack propagation, the whisker pull-out mechanism, and the bridging principle. In addition, the thermal stabilities of the obtained PI-SiCw nanocomposites were improved to a certain extent, and the glass transition temperature was increased. Therefore, in future applications, the addition of SiCw can be fine-tuned according to the actual application of the material, thus improving the applicability of PI nanocomposites.

## Figures and Tables

**Table 1:** Mechanical properties of PI and PI-SiCw nanocomposites.

	Tensile Strength (MPa)	Impact Strength (KJ/m^2^)	Bending Strength (MPa)	Rockwell Hardness (HRR)	Tensile Elongation at Break (%)	Bending Elongation at Break (%)
PI	70.56 ± 1.63	9.66 ± 0.12	78.35 ± 1.91	125.55 ± 0.75	15.52 ± 0.79	3.49 ± 0.48
PI-SiCw-0.5	85.55 ± 1.15	14.37 ± 1.71	104.45 ± 4.50	119.21 ± 0.54	21.61 ± 1.31	8.64 ± 0.79
PI-SiCw-1	89.05 ± 2.35	16.65 ± 3.55	85.25 ± 1.63	120.01 ± 0.61	19.38 ± 0.17	6.50 ± 0.16
PI-SiCw-2	78.60 ± 4.30	24.52 ± 0.73	82.05 ± 5.59	120.03 ± 0.55	18.36 ± 1.21	5.61 ± 0.18
PI-SiCw-5	57.18 ± 5.72	14.76 ± 0.74	62.65 ± 7.57	119.78 ± 0.54	17.37 ± 1.03	3.77 ± 0.25

**Table 2:** TGA and DTG test data of PI and PI-SiCw nanocomposites; T_max_: Maximum decomposition rate temperature; T_50%_: Decomposition temperature at 50% weight loss).

	T_max_ (°C)	T_50%_ (°C)
PI	453.50	594.85
PI-SiCw-0.5	454.47	641.47
PI-SiCw-1	453.00	649.40
PI-SiCw-2	453.46	688.30
PI-SiCw-5	453.40	750.90

## Data Availability

The data used to support this study are included within the article.
